# Perspective of People Living With HIV and Healthcare Workers on the Uptake, Barriers, and Benefits of Multimonth Dispensing: A Qualitative Systematic Review

**DOI:** 10.1155/arat/1392259

**Published:** 2026-05-24

**Authors:** Chioma Wisdom Okezie, Oluwaseun Abdulganiyu Badru, Faith Adeyeoluwa Ibitoye, Joy Chioma Edeh, Oluwafemi Atanda Adeagbo

**Affiliations:** ^1^ The University of Iowa Department of Epidemiology, Iowa City, Iowa, USA; ^2^ The University of Iowa Department of Community and Behavioral Health, Iowa City, Iowa, USA; ^3^ Ubiquitous Educational Consult Limited, Akoka, Lagos, Nigeria

**Keywords:** ARV drug, HIV, multimonth dispensing, multimonth scripting, qualitative study

## Abstract

**Introduction:**

Multimonth dispensing (MMD) is a strategy in the HIV care continuum for people living with HIV (PLWH), especially for those who are virally suppressed. With the increase in MMD following the COVID‐19 pandemic, there is a dearth of data on its impact on HIV care outcomes, such as viral suppression. Therefore, we conducted a qualitative systematic review to explore how PLWH and healthcare workers (HCWs) perceive the uptake, barriers, challenges, and benefits of MMD, as well as its effects on viral suppression.

**Methods:**

In January 2025, following the PRISMA approach, we searched CINAHL, Embase, PubMed, and Scopus databases for articles. Two reviewers independently performed the screen, extraction, and appraisal processes. We descriptively reported the findings in line with our objectives.

**Results:**

Of the 3521 studies found, only 15 were included in this review, and most were from sub‐Saharan Africa. HCWs initiated PLWH on MMD because of the COVID‐19 pandemic, particularly to reduce clinic traffic, even when they did not meet the criteria for MMD. The barriers to PLWH initiating MMD, confirmed by HCWs, include privacy concerns and the stigma associated with having multiple antiretroviral therapy (ART) medication bottles and the stockout of ART medications in clinics. Furthermore, some PLWH refused MMD because plenty of ART bottles can increase the risk of unintended HIV disclosure. Confirmed by HCWs, PLWH share their medication with others and, at times, misuse it. Regarding MMD benefits, PLWH reported job stability as a benefit because of reduced permission from work to refill ART medication and waiting time in the clinics, a decrease in stigma and discrimination, and a generally improved HIV care experience; all confirmed by HCWs. Furthermore, HCWs reported benefits, including reduced workload and burnout. Interestingly, unlike PLWH’s claim that MMD improved adherence and viral suppression, HCWs reported the opposite.

**Conclusion:**

The COVID‐19 pandemic increased MMD rollout to those who met and those who did not meet its criteria, leading to shorter waiting times, job stability, and reduced HCWs’ burnout. However, HIV clinics should initiate MMD for PLWH who meet the criteria, which allows for closer monitoring of the unsuppressed PLWH.

## 1. Introduction

Antiretroviral therapy (ART) has become a globally recommended standard of care for all people living with HIV (PLWH), irrespective of their clinical or subclinical status, to reduce HIV‐related morbidity and mortality [1]. Universal access to ART has transformed HIV into a manageable chronic condition, allowing PLWH to live longer and healthier lives [2]. However, sustaining this success depends on consistent engagement with healthcare systems for medication refills and clinical monitoring. In many low‐ and middle‐income countries (LMICs), overburdened facilities, staff shortages, and the time and financial costs of frequent visits continue to pose major challenges for both patients and healthcare workers (HCWs) [3, 4]. Due to these challenges faced by both PLWH and HCWs, many HIV clinics introduced differentiated service delivery (DSD) approaches to enhance adherence to ART and strengthen engagement in HIV care by minimizing the time and financial burdens placed on clients [5]. Within this framework, multimonth dispensing (MMD) of ART is employed as a service delivery strategy that extends medication refill intervals, improving convenience for clinically stable clients and supporting more efficient use of health system resources. It was recommended by the World Health Organization (WHO) in 2016 [6]. MMD is especially for clients who are aged ≥ 5 years who have been on first‐line ART for at least 6 months, demonstrate consistent adherence, have a suppressed viral load, show no evidence of opportunistic infections, and are not pregnant or breastfeeding [7, 8].

Furthermore, the MMD model for ART allows clinically stable clients to receive a 3‐ to 6‐month supply, or longer in select cases, thereby reducing the need for frequent clinic visits and associated costs [9]. This approach has been shown to improve convenience and satisfaction among clients, while reducing congestion in health facilities and easing the workload of HCWs. By minimizing the frequency of facility visits, MMD supports sustained engagement in care and promotes adherence to treatment, particularly in resource‐limited settings where structural barriers, such as transportation costs and long waiting times, are common [1, 4]. For adult participants, eligibility also required documented viral load suppression within 6 months before study entry and adherence to scheduled clinic appointments, ensuring that only clients demonstrating treatment stability were enrolled in this model. The onset of the COVID‐19 pandemic further magnified existing challenges in accessing routine HIV care, disrupting service delivery, and threatening to reverse progress in HIV treatment outcomes achieved over the past decade [10, 11].

In response to several COVID‐19 pandemic restrictions (including movement from one point to another), many countries accelerated the adoption of MMD, typically providing 3‐ to 6‐month ART refills for clinically stable patients with sustained viral suppression [12, 13]. This adaptation not only reduced facility congestion and patient exposure risk during the pandemic but also reinforced MMD as a key strategy for maintaining continuity of HIV care in resource‐limited settings. However, the COVID‐19 pandemic also prompted the extension of MMD to certain patients who were not yet clinically stable or virally suppressed, a deviation that reflected emergency‐driven flexibility rather than an intentional redefinition of eligibility [14].

Although other quantitative studies have demonstrated the effectiveness of MMD in improving adherence and maintaining viral suppression [15, 16], the successful implementation and sustainability of MMD depend strongly on the experiences, perceptions, and contextual realities of both PLWH and HCWs. Evidence on MMD from the lived experiences of PLWH and HCWs exists [1, 4, 17, 18], but no prior qualitative systematic review has comprehensively synthesized the perspectives of both groups on uptake, barriers, challenges, and perceived benefits of MMD. As such, this systematic review aimed to comprehensively synthesize qualitative evidence on the perceptions of PLWH and HCWs regarding the implementation of MMD of ART. In addition, this review assessed how MMD may influence adherence to ART and viral suppression outcomes and provided context for future research and policy interventions aimed at optimizing DSD models for HIV care.

## 2. Methods

Our review followed the Updated Systematic Review and Meta‐Analysis Protocol (PRISMA‐P; see Appendix 1 [see Table A1] for the PRISMA checklist) in conducting this review [19, 20] and was registered on PROSPERO (CRD420261361148).

### 2.1. Eligibility Criteria

We included only primary qualitative studies that explored the experiences, perceptions, attitudes, and perspectives of PLWH and/or HCWs regarding the uptake, barriers, challenges, and benefits of ART MMD. We considered studies conducted in all geographical settings. Eligible studies involved participants diagnosed with HIV and/or HCWs engaged in HIV service delivery and specifically focused on MMD or extended ART refill models within differentiated HIV care. However, we excluded studies consisting solely of secondary evidence, such as systematic or narrative reviews, commentaries, or editorials, because they do not provide primary empirical data. We also checked existing similar reviews for relevant studies.

### 2.2. Database and Search Strategy

We searched four major health‐related electronic databases: PubMed, CINAHL, Scopus, and Embase in January 2025; two reviewers (CWO and OAB) conducted the systematic search. The search strategy combined both keywords and Medical Subject Headings (MeSH) terms to maximize the retrieval of relevant studies. Specifically, we incorporated three core (i.e., HIV, MMD, and qualitative studies) concept domains: (HIV OR “HIV”[Mesh] Acquired Immunodeficiency Syndrome [Mesh] OR AIDS OR HIV‐positive OR HIV‐Infected OR PLWH OR PLHIV OR “Human Immunodeficiency Virus” OR HIV/AIDS OR antiretroviral∗ OR “Acquired Immunodeficiency Syndrome” OR “Acquired Immune Deficiency Syndrome Virus” OR “Acquired Immunodeficiency Syndrome Virus” OR “Acquired Immunologic Deficiency Syndrome” OR “Acquired Immune Deficiency Syndrome” OR “Acquired Immuno‐Deficiency Syndrome” OR “Acquired Immunodeficiency” OR “HIV seropositivity” OR “HIV seroprevalence”) AND (Multi‐month OR multimonth, “multimonth Dispensing” OR “multimonth suppl∗” OR “multi‐month Dispensing” OR “Prolonged dispensing” OR “Multi‐month prescription∗” OR “Multiple‐month dispensing” OR “Multi‐Month Antiretroviral Therapy Dispensing” OR “Multimonth Antiretroviral Therapy Dispensing” OR MMD OR 3MMD OR 3‐MMD OR 6MMD OR 6‐MMD OR “Antiretroviral Therapy” OR ART OR “ARV Drug∗” OR “Antiretroviral Drugs”) AND (“Qualitative study” OR “Qualitative research” OR “Qualitative analysis” OR Ethnography OR “Grounded Theory” OR “Narrative study” OR Phenomenology OR mixed‐method∗). See full search strategy in Appendix 2 (see Table A2).

Following completion of the electronic database search, we reviewed the reference lists of the included studies and searched Google and Google Scholar for additional relevant empirical articles. We did not limit our search by language or date. We then downloaded the search output from the databases and managed them in the Rayyan systematic review manager [21].

### 2.3. Article Selection Process and Data Extraction

Our first step in the article selection process was deduplication, conducted by one reviewer (CWO). Then, in accordance with the PRISMA guidelines (see Appendix 1), two reviewers (CWO and OAB) independently screened all the article titles and abstracts. We considered studies deemed potentially eligible for full‐text screening, and both reviewers independently conducted the screening. For both the abstract and full‐text screening, the reviewers met to discuss discrepancies, which were resolved without the need for a third reviewer.

Regarding data extraction, two reviewers (CWO and OAB) independently extracted data from the studies. Adapted from previous reviews [22–24], we extracted the following information for each study: authors and publication year, country, data collection type (e.g., in‐depth interview or focus groups), study population (PLWH, HCWs, or both), sample size, sampling technique, year data was obtained, type of MMD (e.g., 3MMD and 6MMD), data analysis (e.g., inductive and deductive), and participants’ characteristics.

### 2.4. Quality Assessment

Using the Critical Appraisal Skills Programme (CASP) for qualitative research [25], we critically appraised the included studies. CASP has 10 items measured with, yes (coded as 1), no, or can’t tell (both coded as 0). The score ranges from 0 to 10, and we considered studies with a score of 7 or more as high quality [25]. Two reviewers (CWO and OAB) independently conducted the appraisal and met to resolve discrepancies.

### 2.5. Data Analysis

We compiled the quotes from the studies and manually coded them to identify common themes. One reviewer experienced in qualitative research (OAB) coded the findings, which were verified by a second reviewer (CWO). We narrated the findings from the analysis descriptively.

## 3. Results

**FIGURE 1:**
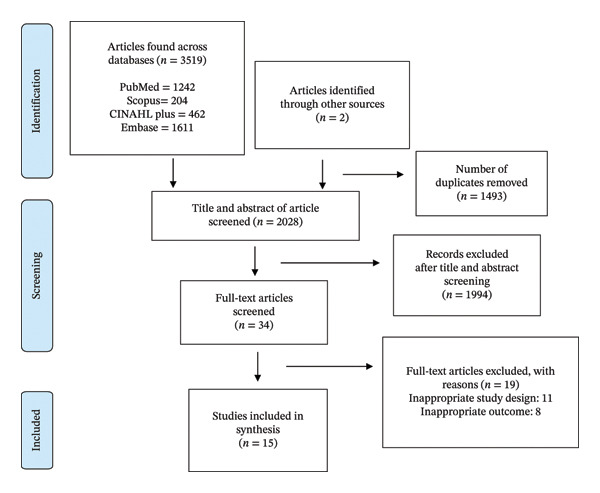
PRISMA flowchart showing study selection for this review.

Of the 3521 articles from the databases and hand search, 15 qualitative studies met our eligibility criteria and were included in this systematic review (Figure 1). The studies were conducted across multiple countries, predominantly in sub‐Saharan Africa (SSA), including Uganda [14, 26–28], Malawi [4, 29], South Africa [17, 30], Ethiopia [18], Nigeria [5], Tanzania [7], Zambia [8], and Zimbabwe [1]; one study was conducted in three SSA countries: Malawi, Tanzania, and South Africa [31]. Only one study was conducted outside SSA, specifically in India [32]. Across the studies, approximately 1295 participants were included, with sample sizes ranging from 14 to 248, including PLWH receiving ART, HCWs involved in MMD delivery, and key stakeholders, such as ART focal persons and program managers.

Data collection methods varied across the studies. Many studies combined in‐depth interviews, focus group discussions, and key informants. Of the 15 studies, 11 used or combined in‐depth interviews [1, 4, 5, 7, 8, 17, 18, 26, 28, 29, 31]. Five studies had elements of key informant interviews [14, 17, 26, 27, 30], and three studies had focus group discussions alone or with another method [18, 29, 32]. Data collection took place in healthcare facilities, with only one study conducted in the community [32]. Moreover, several studies were conducted during the COVID‐19 pandemic [14, 26, 28, 30].

Eleven of the 15 studies conducted an inductive qualitative analysis [1, 7, 8, 14, 17, 27–32]. Two studies used the deductive approach; one study deductively analyzed data into the Andersen Behavioral Model [5], while the other study used the socioecological model [4]. The remaining two studies combined inductive and deductive approaches [18, 26]. A summary of the included studies is provided in Table 1.

**TABLE 1:** Characteristics of the included studies.

S/N	Author	Country	Data collection mode (focus groups or in‐depth interviews or key informant interviews) and location (facility or community) and period (COVID or not)	Study population and sample size	Study sampling and year of data collection (purposive or respondent‐driven sampling)	Multimonth period (3 MMD or 6 MMD)	Data analysis (deductive or inductive)	Participant’s characteristics (gender and average age: mean [SD] or median [IQR] or modal age group or range)	Evidence level
1	Izudi et al. [14]	Uganda	6 PHC facilities 14 KIIs (4 ART focal persons and 10 lay health workers) COVID‐19‐induced MMD	14 HCWs	Purposive sampling between February and March 2022	3 MMD or more (5 months maximum)	Inductive	HCWs Mean age: 34.4 (no SD) Female: 11 (78.6%) Male: 3 (21.4%)	High

2	Du Toit et al. [1]	Zimbabwe	20 public health facilities 20 focus groups (10 from 3 MMD, 10 from 6 MMD) 20 IDIs with HCWs	158 PLWH and HCWs	Purposive sampling between April and June 2019	3 MMD and 6 MMD	Inductive	PLWH Age range: 22–65 Female: 114 (72.2%) Male: 44 (27.8%)	High

3	Akatukwasa et al. [26]	Uganda	2 HIV facilities 24 IDIs (PLWH: 20; HCWs: 4) 6 KIIs COVID‐19‐induced MMD	30 PLWH and HCWs	Purposive sampling between January and March 2022	3–6 MMD	Inductive and deductive	HCWs Male 2: (50%) Female: 2 (50%) Healthcare managers Median age: 46 (36–54) Male: 4 (66.7%) Female: 2 (33.3%) PLWH Median age: 34 (18–51) Male: 10 (50%) Female: 10 (50%)	High

4	Semo et al. [5]	Nigeria (mixed methods)	16 health facilities 79 IDIs (PLWH: 40; HCWs: 39) 6 focus groups with HCWs	79 PLWH and HCWs	Purposive sampling between April 2021 and March 2022	6 MMD	Deductive into the Andersen Behavioral Model	HCWs Median age: 39 (30–35) Female: 19 (49%) Male: 20 (51%) PLWH Median age: 39 (24–43) Female: 18 (45%) Male 22: (55%)	High

5	Machumu et al. [7]	Tanzania	3 health facilities 22 IDIs (PLWH: 13; HCWs: 9)	22 PLWH and HCWs	Purposive sampling in June 2021	3–6 months	Inductive	HCWs Modal age group 25–34: 4 (44%) Female: 5 (67%) Male: 4 (44%) PLWH Modal age group 35–44: 5 (38%) Female: 11 (85%) Male: 2 (15%)	High

6	Tlhaku et al. [30]	South Africa	9 public health clinics and 2 key stakeholder organizations 21 KIIs COVID‐19‐induced MMD	21 HCWs	Purposive and snowball samplings between February and December 2022	3–12 MMD	Inductive	HCWs Female: 16 (76.2%) Male: 5 (23.8%)	High

7	Ware et al. [28]	Uganda	12 HIV clinics IDIs (serodiscordant couples: 25; HCWs: 35) COVID‐19‐induced MMD	60 PLWH and HCWs	Purposive sampling between September 2018 and July 2021	3 MMD	Inductive	HCWs Median age: 35 (31–42.5) Female: 28 (80%) Male 7: (20%) PLWH (couples) Median age: 29 (25–31) Female: 12 (48%) Male: 13 (52%)	High

8	Mantell et al. [18]	Ethiopia	3 facilities 22 IDIs with HCWs 12 FGDs with PLWH (*n* = 93)	115 PLWH and HCWs	Purposive sampling in September 2019	6 MMD	Inductive and deductive (framework not stated)	HCWs Median age: 39 years (34–42) Female: 8 (82%) Male: 4 (18%) PLWH Median age: 41.5 years (36–48) Female: 48 (52%) Male: 45 (48%)	High

9	Chimukuche et al. [31]	Multicountry (Tanzania, Malawi, and South Africa)	34 facilities (South Africa = 17; Malawi = 5, and Tanzania = 12) 87 IDIs with PLWH	87	Purposive sampling between 2018 and 2019	3 MMD or more	Inductive	—	High

10	Keene et al. [17]	South Africa	6 KIIs 30 IDIs (PLWH: 23; HCWs: 7)	36	Purposive sampling between 2017 and 2019	2–6 MMD	Inductive	HCWs Age range: 31–59 Female: 6 (85.7%) Male: 1 (14.3%) Key informants Age range: not provided Female: 3 (50.0%) Male: 3 (50.0%) PLWH Age range: 30–66 Female: 21 (91.3%) Male: 2 (8.7%)	High

11	Prust et al. [29]	Malawi (mixed methods)	30 facilities 32 IDIs with HCWs 30 FGDs with PLWH (*n* = 216)	248	Purposive sampling between February and May 2016	3 MMD	Inductive	PLWH Modal age group: 31–45: 102 (47.7%) Male: 93 (43.1%) Female: 123 (56.9%)	High

12	Phiri et al. [8]	Zambia	10 facilities 18 IDIs with HCWs	18	Convenience sampling between July and August 2018	3–6 MMD	Inductive	HCWs Modal age: 25–34 (38.9%)	High

13	Pollard et al. [32]	India	Communities (in two states) 7 FGD (5‐8 participants per FGD) with MSM, FSW, and TGW	44	Purposive sampling between November and December 2020	3 MMD	Inductive	PLWH Median age: 31: 20–49	High

14	Kabwama et al. [27]	Uganda (qualitative with review)	17 facilities 21 KIIs with HCWs	21	Purposive sampling in 2020	3–6 MMD	Inductive	—	High

15	Hubbard et al. [4]	Malawi	10 facilities 79 IDIs (PLWH: 62; HCWs: 17)	79	Random sampling between June and August 2018	3–6 MMD	Deductive into the socioecological model	HCWs Median age: 35 years Female: 9 (52.9%) Male: 8 (47.1) PLWH Median age: 41.5 (no IQR) Female: 32 (51.6%) Male: 30 (48.4%)	High

*Note:* MMD: multimonth dispensing; TGW: transgender women; MSM: men who have sex with men.

Abbreviations: FGD, focus group discussion; FSW, female sex workers; KII, key informant interview; PLWH, people living with HIV.

Regarding the quality of the included studies, our analysis rated them all as high quality. However, 11 of the 15 studies checked nine of the CASP 10 items [4, 5, 8, 14, 18, 26–31]. These studies did not report on the relationship between the interviewer and the participants, nor how it may have influenced the data. One study checked eight of the 10 items, also missing information on the relationship between the interviewers and interviewees, and there was no evidence of ethical considerations [1]. Three studies checked all 10 items of the CASP appraisal tool [7, 17, 32]. See the appraisal checklist in Appendix 3 (see Table A3).

### 3.1. MMD Uptake

Across studies, the initiation of MMD was shaped by both patient needs and health system priorities. For example, evidence shows that some PLWH requested MMD to accommodate their distance to the health facility and long travels to reduce the burden of frequent clinic visits [31], while in some cases, HCWs initiate MMD for PLWH to reduce queues and overcrowding in the health facility [7]. HCWs utilized this system‐level strategy to reduce clinic congestion, manage patient volumes, and improve service delivery efficiency, as evidenced in Malawi [4, 29]. Moreover, HCWs were prompted to start PLWH on MMD because of COVID‐19, even when they do not meet MMD criteria [14, 30], and in some cases, PLWH demand MMD because of COVID‐19 risks [14].

### 3.2. MMD Benefits

**FIGURE 2:**
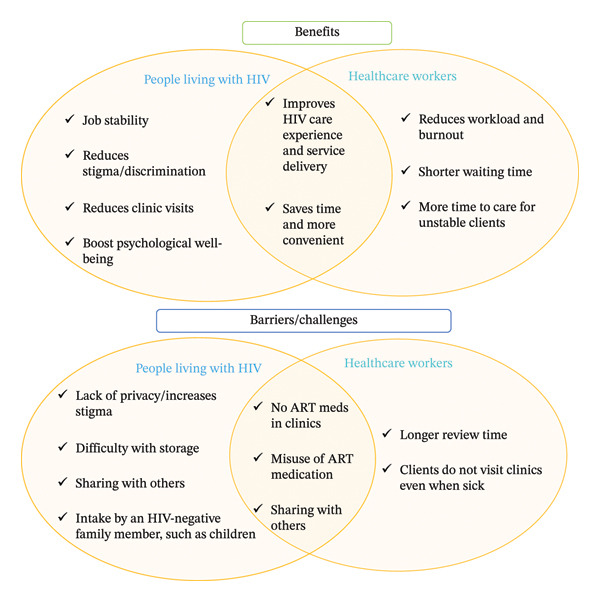
Benefits, barriers, and facilitators to MMD from the perspective of people living with HIV and healthcare workers.

In Figure 2, we showed the distinct and overlapping benefits and barriers/challenges to MMD, as reported by PLWH and HCWs. PLWH consistently described MMD as beneficial. Specifically, they reported that MMD has reduced clinic visits and stress associated with frequent facility visits, especially transportation costs [1, 5, 17, 28, 29], and that MMD allowed them to do other things instead of recurrent clinic visits [1, 4]. HCWs alluded to the reduced frequency of visits to the health facility and how this saves time [1, 5, 8, 18, 27, 30] and transportation fare for clients [8, 26, 28]. Furthermore, PLWH reported that reduced clinic visit frequency due to MMD enabled greater participation in work or daily activities and guaranteed job stability and security [1, 4, 5, 7, 17, 28, 29]. In addition, many PLWH reported feeling happier, less worried, and more motivated, with an overall improvement in HIV care experience and services [17, 18]. The HCWs shared these views as well [1, 17, 18]. Furthermore, PLWH reiterated that MMD has helped to reduce their fear of stigma and discrimination, particularly in work settings [17, 18].

Additionally, confirming the perception of PLWH, HCWs reported that MMD has reduced waiting times in the clinic [1, 5, 8, 18, 27, 30] and, more importantly, reduced their workload and burnout [1, 18], thereby affording them more time to attend to more critically sick or unstable PLWH [18, 27, 30].

### 3.3. MMD Barriers and Challenges

Despite the reported benefits of MMD, both PLWH and HCWs reported important barriers and challenges (Figure 2). Specifically, confirmed by HCWs [4], PLWH cited difficulties in storing and concealing multiple bottles of ART (especially when they are provided with six bottles of ART medications) [4, 18, 32], with concerns that it can lead to unintended HIV disclosure if bottles are detected by family or friends [7, 17, 18]. They feared that this would lead to HIV‐related stigma [1, 4]. Also, in some cases, PLWH share ART medication with other clients [4] and sometimes misuse the medication, including adding it to chicken feed or beer, or allowing an HIV‐negative person, such as children, to use it [4, 18]. HCWs confirmed these misuses of ART following MMD [4, 8].

Furthermore, HCWs highlighted systemic challenges of ART medication shortages [7, 17, 30]. In addition, HCWs stressed the challenge of following up with PLWH on MMD, with many PLWH taking longer than expected to return to the health facility for follow‐up, even when they are sick before their ART refill due date [4, 8, 30].

### 3.4. Adherence and Viral Suppression

The perception of MMD influence on adherence to ART medication and viral suppression differs considerably between PLWH and HCWs. There is convergence on the positive influence of MMD on ART adherence from the PLWH and HCWs’ perspectives [5, 29]. However, there is also disagreement between both groups, with PLWH indicating that it helps maintain ART adherence [5, 8], and HCWs reporting lack of adherence to ART medication [5, 7, 29, 30], with some PLWH being returned to a monthly refill due to lack of adherence to ART medication [7]. Similarly, some studies reported a positive influence of MMD on viral suppression for both PLWH [5] and HCWs [17], whereas some HCWs reported viral rebound following MMD initiation and difficulty bringing PLWH in for blood work [30].

## 4. Discussion

This review examined the perspectives of PLWH and HCWs on the uptake, benefits, and challenges of MMD. This review identified nine countries that have documented their experiences with MMD since it started in 2016. This highlights the need for additional qualitative studies in countries that have implemented MMD to better understand the barriers and facilitators to its implementation among PLWH. Our main findings are that PLWH and HCWs consider MMD as a useful strategy to reduce workload in HIV clinics and reduce waiting times for PLWH. The challenges with MMD include misuse of ART, compromised privacy due to possession of many medication bottles, and extended delays in returning to HIV clinics, as well as mixed reactions to its influence on adherence and viral suppression.

In this review, we found that only four of the 15 studies explained the reasons for adopting MMD. The findings showed that MMD uptake was shaped by the desire to reduce the frequency of visits and clinic contacts, as well as the need to tailor medications to individual preferences [14, 31]. Administering medications according to patients’ preferences has become a common strategy, as reported by Belay et al. [33], to inform and improve clinical and policy decision‐making, especially in resource‐limited settings. However, during the pandemic, Tlhaku et al. [30] noted that MMD uptake was primarily among clinically stable clients, consistent with WHO guidance, even during the COVID‐19 pandemic. MMD implementation among other client groups is an emerging strategy necessitating further research.

Nearly all the studies (13 of 15) reported that the MMD was beneficial. The reasons cited were consistent across these studies, including having more time to engage in work and family activities, saving more, planning extended travels, experiencing less fear of discrimination, and generally living a happy life. HCWs also confirmed these advantages, noting that this approach enhanced their work efficiency and shortened wait times in the clinic. Another review supports some of these findings, including reductions in transportation, direct medical expenses, and childcare costs, which are linked to lower treatment adherence [33]. Therefore, our findings suggest that MMD offers PLWH an opportunity to reduce expenses, save more, maintain their treatment adherence, and support basic household needs.

Furthermore, we found that while most studies reported benefits of MMD, some HCWs expressed concerns about missed appointments and reduced follow‐up, and alluded to the fact that MMD may have contributed to nonadherence and loss of viral suppression. Conversely, PLWH generally maintained adherence and viral suppression. However, PLWH did not report nonadherence or loss of viral suppression following MMD initiation. These divergent findings call for more investigation as to why there is disagreement between PLWH and HCWs, particularly because many HIV clinics provide MMD.

Another important barrier worth discussing is the stockout of ART medication in health facilities. MMD is only sustainable if there is enough medication to sustain the routine. Research has found that medication stockouts in HIV clinics can lead patients to discontinue treatment or HCWs to prescribe alternatives [34]. Unplanned treatment breaks may allow the virus to mutate and develop resistance, reducing the effectiveness of future treatments [34]. With the recent cut in HIV funding, some facilities (particularly those in low‐resource settings) may find it harder to sustain MMD for PLWH, with associated benefits, such as job stability and reduction in stigma and discrimination, threatened. We recommend, where possible, that HIV clinics maintain proper inventory and strategic plans, establish a robust supply chain, and make operational adjustments, such as switching to an alternative regimen in accordance with the country’s national guidelines [35, 36]. Furthermore, HIV clinics, as done in Tanzania, can borrow inventory from other facilities, and in extreme cases, transfer PLWH to nearby facilities that are convenient for them, to maintain PLWH’s regimen [37].

This review has both strengths and limitations. Nearly all the studies in this review originated from SSA, limiting findings to the region. Moreover, the focus on qualitative studies only implies that our review shares the limitation of qualitative studies; that is, that findings cannot be generalized. Also, because we reanalyzed the quotes from the studies, there is a possibility of losing contextual meaning. Lastly, we cannot rule out the possibility of missing some papers despite our extensive probe of the literature. Nonetheless, this review offers insights into the challenges of MMD, its benefits for PLWH and HCWs, and its implications for achieving Sustainable Development Goal 3.3 (ending the AIDS epidemic).

## 5. Conclusions

Overall, findings from the review indicate that MMD is widely perceived by both PLWH and HCWs as beneficial and acceptable, with its rationale and implementation largely consistent with WHO guidance for clinically stable patients. PLWH opined that MMD provides them with job stability and helps reduce stigma/discrimination that often arises from frequent encounters with HIV clinics. They also mentioned that MMD goes beyond saving them time for other things, such as frequent visits to HIV clinics, and improves their HIV care experiences and boosts their psychological well‐being. From the perspective of the HCWs, MMD decreases their workload and, consequently, their burnout. It also allows more time to care for unstable or more critically ill PLWH with complex needs.

PLWH and HCWs highlighted barriers and challenges related to MMD. PLWH worried about medication storage, fewer counseling opportunities, and clinical contact, especially with longer dispensing intervals. HCWs cited structural issues, such as ART stockouts, administrative burdens, limited monitoring between visits, and concerns about medication sharing, misuse, and loss. Perceptions of adherence and viral suppression varied: PLWH saw MMD as supporting adherence and viral suppression, whereas HCWs had mixed views due to less clinical contact and delayed laboratory results. These findings emphasize the need for improved ART supply chains, harmonized guidelines for viral suppression and MMD eligibility, and balanced monitoring approaches.

## Funding

The authors did not receive funding for this project.

## Ethics Statement

The authors have nothing to report.

## Conflicts of Interest

The authors declare no conflicts of interest.

## Data Availability

The data that support the findings of this study are available from the corresponding author upon reasonable request.

## Appendix 1 PRISMA checklist

**TABLE A1:** PRISMA checklist.

Section and topic	Item #	Checklist item	Location where item is reported
*Title*			
Title	1	Identify the report as a systematic review.	1

*Abstract*			
Abstract	2	See the PRISMA 2020 for Abstracts checklist.	2

*Introduction*			
Rationale	3	Describe the rationale for the review in the context of existing knowledge.	4
Objectives	4	Provide an explicit statement of the objective(s) or question(s) the review addresses.	4

*Methods*			
Eligibility criteria	5	Specify the inclusion and exclusion criteria for the review and how studies were grouped for the syntheses.	4‐5
Information sources	6	Specify all databases, registers, websites, organizations, reference lists, and other sources searched or consulted to identify studies. Specify the date when each source was last searched or consulted.	5
Search strategy	7	Present the full search strategies for all databases, registers, and websites, including any filters and limits used.	5
Selection process	8	Specify the methods used to decide whether a study met the inclusion criteria of the review, including how many reviewers screened each record and each report retrieved, whether they worked independently, and if applicable, details of automation tools used in the process.	5‐6
Data collection process	9	Specify the methods used to collect data from reports, including how many reviewers collected data from each report, whether they worked independently, any processes for obtaining or confirming data from study investigators, and if applicable, details of automation tools used in the process.	5‐6
Data items	10a	List and define all outcomes for which data were sought. Specify whether all results that were compatible with each outcome domain in each study were sought (e.g., for all measures, time points, and analyses), and if not, the methods used to decide which results to collect.	6
	10b	List and define all other variables for which data were sought (e.g., participant and intervention characteristics, funding sources). Describe any assumptions made about any missing or unclear information.	6
Study risk‐of‐bias assessment	11	Specify the methods used to assess risk of bias in the included studies, including details of the tool(s) used, how many reviewers assessed each study and whether they worked independently, and if applicable, details of automation tools used in the process.	6
Effect measures	12	Specify for each outcome the effect measure(s) (e.g., risk ratio and mean difference) used in the synthesis or presentation of results.	N/A
Synthesis methods	13a	Describe the processes used to decide which studies were eligible for each synthesis (e.g., tabulating the study intervention characteristics and comparing against the planned groups for each synthesis (item #5)).	6
	13b	Describe any methods required to prepare the data for presentation or synthesis, such as handling of missing summary statistics, or data conversions.	N/A
	13c	Describe any methods used to tabulate or visually display results of individual studies and syntheses.	6
	13d	Describe any methods used to synthesize results and provide a rationale for the choice(s). If meta‐analysis was performed, describe the model(s), method(s) to identify the presence and extent of statistical heterogeneity, and software package(s) used.	6
	13e	Describe any methods used to explore possible causes of heterogeneity among study results (e.g., subgroup analysis and meta‐regression).	N/A
	13f	Describe any sensitivity analyses conducted to assess robustness of the synthesized results.	N/A
Reporting bias assessment	14	Describe any methods used to assess risk of bias due to missing results in a synthesis (arising from reporting biases).	6
Certainty assessment	15	Describe any methods used to assess certainty (or confidence) in the body of evidence for an outcome.	6

*Results*			
Study selection	16a	Describe the results of the search and selection process, from the number of records identified in the search to the number of studies included in the review, ideally using a flow diagram.	6
	16b	Cite studies that might appear to meet the inclusion criteria, but which were excluded, and explain why they were excluded.	N/A
Study characteristics	17	Cite each included study and present its characteristics.	7
Risk of bias in studies	18	Present assessments of risk of bias for each included study.	7
Results of individual studies	19	For all outcomes, present, for each study: (a) summary statistics for each group (where appropriate) and (b) an effect estimate and its precision (e.g., confidence/credible interval), ideally using structured tables or plots.	N/A
Results of syntheses	20a	For each synthesis, briefly summarize the characteristics and risk of bias among contributing studies.	7‐8
	20b	Present results of all statistical syntheses conducted. If meta‐analysis was done, present for each the summary estimate and its precision (e.g., confidence/credible interval) and measures of statistical heterogeneity. If comparing groups, describe the direction of the effect.	N/A
	20c	Present results of all investigations of possible causes of heterogeneity among study results.	N/A
	20d	Present results of all sensitivity analyses conducted to assess the robustness of the synthesized results.	N/A
Reporting biases	21	Present assessments of risk of bias due to missing results (arising from reporting biases) for each synthesis assessed.	7
Certainty of evidence	22	Present assessments of certainty (or confidence) in the body of evidence for each outcome assessed.	7

*Discussion*			
Discussion	23a	Provide a general interpretation of the results in the context of other evidence.	9
	23b	Discuss any limitations of the evidence included in the review.	10‐11
	23c	Discuss any limitations of the review processes used.	10‐11
	23d	Discuss implications of the results for practice, policy, and future research.	11

*Other Information*			
Registration and protocol	24a	Provide registration information for the review, including register name and registration number, or state that the review was not registered.	4
	24b	Indicate where the review protocol can be accessed, or state that a protocol was not prepared.	N/A
	24c	Describe and explain any amendments to information provided at registration or in the protocol.	N/A
Support	25	Describe sources of financial or nonfinancial support for the review, and the role of the funders or sponsors in the review.	1
Competing interests	26	Declare any competing interests of review authors.	1
Availability of data, code, and other materials	27	Report which of the following are publicly available and where they can be found: template data collection forms; data extracted from included studies; data used for all analyses; analytic code; any other materials used in the review.	15–19

## Appendix 2 Search Strategies

**TABLE A2:** Search strategies.

*PUBMED*	
HIV OR “HIV”[Mesh] Acquired Immunodeficiency Syndrome [Mesh] OR AIDS OR HIV‐POSITIVE OR HIV‐Infected OR PLWH OR PLHIV OR “Human Immunodeficiency Virus” OR HIV/AIDS OR antiretroviral∗ OR “Acquired Immunodeficiency Syndrome” OR “Acquired Immune Deficiency Syndrome Virus” OR “Acquired Immunodeficiency Syndrome Virus” OR “Acquired Immunologic Deficiency Syndrome” OR “Acquired Immune Deficiency Syndrome” OR “Acquired Immuno‐Deficiency Syndrome” OR “Acquired Immunodeficiency” OR “HIV seropositivity” OR “HIV seroprevalence”	473,090
MULTI‐MONTH OR MULTIMONTH, “MULTIMONTH Dispensing” OR “MULTIMONTH suppl∗” OR “MULTI‐MONTH Dispensing” OR “Prolonged dispensing” OR “Multi‐month prescription∗” OR “Multiple‐month dispensing” OR “Multi‐Month Antiretroviral Therapy Dispensing” OR “Multimonth Antiretroviral Therapy Dispensing” OR MMD OR 3MMD OR 3‐MMD OR 6MMD OR 6‐MMD OR “Antiretroviral Therapy” OR ART OR “ARV` Drug∗” OR “Antiretroviral Drugs”	278,151
“QUALITATIVE STUDY” OR “Qualitative Research”[Mesh] OR “Qualitative research” OR “Qualitative analysis” OR Ethnography OR “Grounded Theory” OR “Narrative study” OR Phenomenology OR mixed‐method∗	423,230
((HIV OR “HIV”[Mesh] Acquired Immunodeficiency Syndrome [Mesh] OR AIDS OR HIV‐POSITIVE OR HIV‐Infected OR PLWH OR PLHIV OR “Human Immunodeficiency Virus” OR HIV/AIDS OR antiretroviral∗ OR “Acquired Immunodeficiency Syndrome” OR “Acquired Immune Deficiency Syndrome Virus” OR “Acquired Immunodeficiency Syndrome Virus” OR “Acquired Immunologic Deficiency Syndrome” OR “Acquired Immune Deficiency Syndrome” OR “Acquired Immuno‐Deficiency Syndrome” OR “Acquired Immunodeficiency” OR “HIV seropositivity” OR “HIV seroprevalence” AND (MULTI‐MONTH OR MULTIMONTH, “MULTIMONTH Dispensing” OR “MULTIMONTH suppl∗” OR “MULTI‐MONTH Dispensing” OR “Prolonged dispensing” OR “Multi‐month prescription∗” OR “Multiple‐month dispensing” OR “Multi‐Month Antiretroviral Therapy Dispensing” OR “Multimonth Antiretroviral Therapy Dispensing” OR MMD OR 3MMD OR 3‐MMD OR 6MMD OR 6‐MMD OR “Antiretroviral Therapy” OR ART OR “ARV Drug∗” OR “Antiretroviral Drugs”) AND (“QUALITATIVE STUDY” OR “Qualitative Research”[Mesh] OR “Qualitative research” OR “Qualitative analysis” OR Ethnography OR “Grounded Theory” OR “Narrative study” OR Phenomenology OR mixed‐method∗) Filters: from 2016–2025	1242

*SCOPUS*	
HIV OR AIDS OR HIV‐POSITIVE OR HIV‐Infected OR PLWH OR PLHIV OR “Human Immunodeficiency Virus” OR HIV/AIDS OR antiretroviral∗ OR “Acquired Immunodeficiency Syndrome” OR “Acquired Immune Deficiency Syndrome Virus” OR “Acquired Immunodeficiency Syndrome Virus” OR “Acquired Immunologic Deficiency Syndrome” OR “Acquired Immune Deficiency Syndrome” OR “Acquired Immuno‐Deficiency Syndrome” OR “Acquired Immunodeficiency” OR “HIV seropositivity” OR “HIV seroprevalence”	1,326,748
MULTI‐MONTH OR MULTIMONTH, “MULTIMONTH Dispensing” OR “MULTIMONTH suppl∗” OR “MULTI‐MONTH Dispensing” OR “Prolonged dispensing” OR “Multi‐month prescription∗” OR “Multiple‐month dispensing” OR “Multi‐Month Antiretroviral Therapy Dispensing” OR “Multimonth Antiretroviral Therapy Dispensing” OR MMD OR 3MMD OR 3‐MMD OR 6MMD OR 6‐MMD OR “Antiretroviral Therapy” OR ART OR “ARV Drug∗” OR “Antiretroviral Drugs”	407
“QUALITATIVE STUDY” OR “Qualitative research” OR “Qualitative analysis” OR Ethnography OR “Grounded Theory” OR “Narrative study” OR Phenomenology OR mixed‐method∗	2,430,164
	204

*CINAHL*	
HIV OR AIDS OR HIV‐POSITIVE OR HIV‐Infected OR PLWH OR PLHIV OR “Human Immunodeficiency Virus” OR HIV/AIDS OR antiretroviral∗ OR “Acquired Immunodeficiency Syndrome” OR “Acquired Immune Deficiency Syndrome Virus” OR “Acquired Immunodeficiency Syndrome Virus” OR “Acquired Immunologic Deficiency Syndrome” OR “Acquired Immune Deficiency Syndrome” OR “Acquired Immuno‐Deficiency Syndrome” OR “Acquired Immunodeficiency” OR “HIV seropositivity” OR “HIV seroprevalence”	172,976
MULTI‐MONTH OR MULTIMONTH, “MULTIMONTH Dispensing” OR “MULTIMONTH suppl∗” OR “MULTI‐MONTH Dispensing” OR “Prolonged dispensing” OR “Multi‐month prescription∗” OR “Multiple‐month dispensing” OR “Multi‐Month Antiretroviral Therapy Dispensing” OR “Multimonth Antiretroviral Therapy Dispensing” OR MMD OR 3MMD OR 3‐MMD OR 6MMD OR 6‐MMD OR “Antiretroviral Therapy” OR ART OR “ARV Drug∗” OR “Antiretroviral Drugs”	65,292
“QUALITATIVE STUDY” OR “Qualitative research” OR “Qualitative analysis” OR Ethnography OR “Grounded Theory” OR “Narrative study” OR Phenomenology OR mixed‐method∗	138,154
S1 AND S2 AND S3	462

*EMBASE*	
HIV OR HIV‐POSITIVE OR HIV‐Infected OR PLWH OR PLHIV OR “Human Immunodeficiency Virus” OR antiretroviral∗ OR “Acquired Immunodeficiency Syndrome” OR “Acquired Immune Deficiency Syndrome Virus” OR “Acquired Immunodeficiency Syndrome Virus” OR “Acquired Immunologic Deficiency Syndrome” OR “Acquired Immune Deficiency Syndrome” OR “Acquired Immuno‐Deficiency Syndrome” OR “Acquired Immunodeficiency” OR “HIV seropositivity” OR “HIV seroprevalence”	1,071,481
MULTI‐MONTH OR MULTIMONTH, “MULTIMONTH Dispensing” OR “MULTIMONTH suppl∗” OR “MULTI‐MONTH Dispensing” OR “Prolonged dispensing” OR “Multi‐month prescription∗” OR “Multiple‐month dispensing” OR “Multi‐Month Antiretroviral Therapy Dispensing” OR “Multimonth Antiretroviral Therapy Dispensing” OR MMD OR 3MMD OR 3‐MMD OR 6MMD OR 6‐MMD OR “Antiretroviral Therapy” OR ART OR “ARV Drug∗” OR “Antiretroviral Drugs”	439,826
“QUALITATIVE STUDY” OR “Qualitative research” OR “Qualitative analysis” OR Ethnography OR “Grounded Theory” OR “Narrative study” OR Phenomenology OR mixed‐method∗	350,929
S1 AND S2 AND S3	1611

## Appendix 3 Quality appraisal of the included studies

**TABLE A3:** Quality appraisal of the included studies.

S/N	Items	Izudi et al.	Du Toit et al.	Akatukwasa et al.	Semo et al.	Machumu et al.	Tlhaku et al.	Ware et al.	Mantell et al.	Chimukuche et al.	Keene et al.	Prust et al.	Phiri et al.	Pollard et al.	Kabwama et al.	Hubbard et al.
1	Was there a clear statement of the aims of the research?	Y	Y	Y	Y	Y	Y	Y	Y	Y	Y	Y	Y	Y	Y	Y
2	Is a qualitative methodology appropriate?	Y	Y	Y	Y	Y	Y	Y	Y	Y	Y	Y	Y	Y	Y	Y
3	Was the research design appropriate to address the aims of the research?	Y	Y	Y	Y	Y	Y	Y	Y	Y	Y	Y	Y	Y	Y	Y
4	Was the recruitment strategy appropriate to the aims of the research?	Y	Y	Y	Y	Y	Y	Y	Y	Y	Y	Y	Y	Y	Y	Y
5	Were the data collected in a way that addressed the research issue?	Y	Y	Y	Y	Y	Y	Y	Y	Y	Y	Y	Y	Y	Y	Y
6	Has the relationship between researcher and participants been adequately considered?	N	N	N	N	Y	N	N	N	N	Y	N	N	Y	N	N
7	Have ethical issues been taken into consideration?	Y	C	Y	Y	Y	Y	Y	Y	Y	Y	Y	Y	Y	Y	Y
8	Was the data analysis sufficiently rigorous?	Y	Y	Y	Y	Y	Y	Y	Y	Y	Y	Y	Y	Y	Y	Y
9	Is there a clear statement of findings?	Y	Y	Y	Y	Y	Y	Y	Y	Y	Y	Y	Y	Y	Y	Y
10	How valuable is the research?	Y	Y	Y	Y	Y	Y	Y	Y	Y	Y	Y	Y	Y	Y	Y
	Score	9/10	8/10	9/10	9/10	10/10	9/10	9/10	9/10	9/10	10/10	9/10	9/10	10/10	9/10	9/10

*Note:* Y: yes; N: no; C: cannot tell.
